# Propensity score-matched analysis of physician-controlled wire-guided cannulation as an effective technique against difficult cannulation in endoscopic retrograde cholangiopancreatography: A retrospective study

**DOI:** 10.1371/journal.pone.0285118

**Published:** 2023-04-28

**Authors:** Han Jo Jeon, Jae Min Lee, Sun Young Yim, Hyuk Soon Choi, Eun Sun Kim, Bora Keum, Yeon Seok Seo, Yoon Tae Jeen, Hoon Jai Chun, Hong Sik Lee

**Affiliations:** Division of Gastroenterology and Hepatology, Department of Internal Medicine, Korea University Anam Hospital, Seoul, Republic of Korea; AORN Cardarelli: Azienda Ospedaliera di Rilievo Nazionale Antonio Cardarelli, ITALY

## Abstract

**Background:**

Advanced endoscopic retrograde cholangiopancreatography (ERCP) cannulation strategies for difficult cases could replace conventional techniques, in which assistants control guidewires. We aimed to compare the safety and efficacy of a new salvage cannulation strategy, physician-controlled wire-guided cannulation (PCWGC), with those of a conventional strategy.

**Methods:**

This retrospective study included patients with naïve papillae who underwent ERCP between January 2018 and December 2020. Patients, divided into two groups, received initial cannulation using a conventional catheter. After failed cannulation, the second attempt used PCWGC and double-guidewire technique (DGT) in the new and conventional strategy groups, respectively. Propensity score-matching (PSM) analysis compared outcomes between groups. Primary outcome included overall success rate, while secondary outcomes included cannulation time, adverse events, and cannulation difficulty subgroup analysis.

**Results:**

The new strategy group comprised 255 (47.6%) of 536 patients who underwent ERCP. The total cannulation success rate was 98.4% (vs. 97.2%, p = 0.318), with similar post-ERCP pancreatitis (PEP) (1.8% vs. 2.4%, p = 0.64) rates. Following 1:1 PSM, 219/438 patients were allocated to both the conventional and new strategy groups, and 46 patients from the difficult cannulation subgroup were distributed evenly among groups. No difference in overall cannulation success rate existed between the groups before (97.2% vs. 98.4%) and after PSM (96.8% vs. 98.2%). The primary cannulation success rate was higher in the conventional strategy group, while the secondary cannulation success rate was higher in the new strategy group. However, the difficult cannulation subgroup PSM results showed that only the salvage cannulation success rate was significant (9/23, 39.1% vs. 18/23, 78.3%, p = 0.007). In the difficult cannulation subgroup, the salvage cannulation success rate for PCWGC was four times higher than DGT (95% confidence interval; 1.129–14.175), with no significant difference in PEP rate (p = 0.571).

**Conclusions:**

PCWGC demonstrated greater efficacy than the conventional salvage technique. PCWGC could be a safe and useful technique, especially for difficult biliary cannulation.

## Introduction

Selective cannulation of the common bile duct is a critical procedure in endoscopic retrograde cholangiopancreatography (ERCP). Although successful cannulation is likely to be achieved after multiple ERCP attempts, endoscopists experienced in ERCP often express difficulty performing bile duct cannulation [1, 2]. Cannulation steps performed with either a catheter or a sphincterotome are used to develop the ERCP technique and delineate the biliary system using contrast media and guidewires. The rate of post-ERCP pancreatitis (PEP) is lower in the wire-guided cannulation method than that in the contrast method. However, excessive guidewire manipulation by inexperienced assistants increases the risk of excessive stimulation of the ampulla and the pancreas, resulting in cannulation failure. Although conventional wire-guided cannulation focuses on the importance of coordination between endoscopists and their assistants, there are some limitations pertaining to the proficiency of the assistants in real-world clinical settings.

Currently, there has been an increase in the availability of equipment, accessories, and endoscopic techniques for successful ERCP in clinical practice. Numerous techniques involving the use of cannulas, papillotomes, guidewires, and pre-cut approaches have increased the success rate of selective bile duct cannulation [3]. Although several cannulation strategies are available, therapeutic endoscopists can develop their own strategies for difficult cases of selective cannulation. These techniques include guidewire-assisted cannulation, double-guidewire cannulation, precutting with a needle-knife sphincterotome, and pancreatic duct stenting [4–7].

These advanced techniques require proficient assistance for complete cannulation. Technical advances in guidewire handling without the aid of an assistant have provided endoscopists with the opportunity to control guidewires on their own. Physician-controlled wire-guided cannulation (PCWGC), also known as single-operator wire-guided cannulation, allows the surgeon to place a guide wire during cannulation without assistance. Currently, this technique has proven to be feasible because the guidewire can be effectively controlled by an individual, and the procedure can be performed effectively without being impacted by the inexperience of an assistant [8]. Relatively little information is available regarding the efficacy and safety of PCWGC. This retrospective study aimed to determine whether PCWGC is a safer and more effective salvage cannulation method than assistant-assisted wire-guided cannulation.

## Materials and methods

### Ethical statement

The study protocol was approved and registered retrospectively by the appropriate institutional review board (IRB number: 2019AN0313). This study was conducted in accordance with the ethical guidelines of the Declaration of Helsinki. Informed consent regarding ERCP was obtained from all individual participants prior to the experiment, and the study was approved by the ethics committee of The Korea University Medical Center. Patient consent was not required for this retrospective study.

### Study participants

This single-center analysis included all patients who underwent ERCP between January 2018 and December 2020 at Korea University Medical Center. Patients who underwent therapeutic ERCP with a naïve ampulla were examined. Anonymized medical records were retrospectively reviewed, and cannulation time, success rate, adverse events, laboratory data, and underlying comorbidities were extracted. Three experienced therapeutic endoscopists performed ERCP at the center. Two well-trained nurses assisted in the manipulation of the guidewires.

### Inclusion and exclusion criteria

This study included patients with naïve ampulla who were candidates for standard biliary cannulation, including those with choledocholithiasis, bile leakage, and biliary obstruction. The exclusion criteria were (1) prior ERCP with sphincterotomy, (2) pregnancy, (3) age <18 years, (4) inability to give informed consent, (5) cannulation protocol violation, (6) prior upper gastrointestinal surgery, (7) cases where only pancreatic duct evaluation was required, (8) presence of pancreatobiliary ectopic openings, (9) allergy to contrast dye, and (10) unclear endoscopic images describing cannulation time.

### ERCP cannulation protocol

At our center, the standard strategy for cannulation until January 2019 was the sequential application of 1) cannulation catheter access, 2) double-guidewire technique (DGT), and 3) precut infundibulotomy. In January 2019, a new strategy was adopted, which included 1) cannulation catheter access, 2) PCWGC, and 3) precut infundibulotomy. In the conventional strategy group, an initial attempt was made to cannulate the papilla using a catheter. In cases where selective cannulation was difficult or failed by standard cannulation, the technique was switched to DGT, in which the guidewire was placed into the pancreatic duct (PD). Subsequently, cannulation was performed using a catheter. If the guidewire failed to be repeatedly inserted into the bile duct, precut infundibulotomy was performed as a rescue technique. The PCWGC method was adopted after January 2019. If cannulation catheter access and PCWGC for selective cannulation failed, the endoscopist performed a final pre-cut infundibulotomy or sphincterotomy.

### ERCP cannulation techniques

#### Conventional assistant-assisted guidewire

Following the intubation of the endoscope into the duodenum, a catheter tip (MTW Endoskopie, Wesel, Germany) was gently inserted through the channel and targeted to the orifice of the ampulla. Subsequently, the catheter tip was inserted into the ampulla to a minimal depth of approximately 2–3 mm. Skillful assistants carefully advanced the guidewire (VisiGlide2 guidewires, Olympus, Tokyo, Japan) into the bile duct under fluoroscopic guidance.

#### Physician-controlled wire-guided cannulation

PCWGC was performed using the following steps: a sphincterotome preloaded with a guidewire (Jagtome RX^™^ guidewire preloaded sphincterotome, Boston Scientific, Natick, MA, USA) was passed through the endoscope and below the major papilla. The guidewire was then “peeled” (removed from the inside of the cannula) just above the operating channel port. A physician gently advanced the guidewire into the papillary orifice and manipulated it into the bile duct under fluoroscopic guidance.

Accessory devices for the ERCP pre-cut techniques included a NeedleCut3V needle knife (Olympus, Tokyo, Japan). ERCP was performed using the Olympus EVIS Lucera 260 Endoscopy System (Olympus Optical, Tokyo, Japan) and Olympus JF-260V or TJF-260V (Olympus Optical). Following the ERCP procedure, laboratory tests, including amylase and lipase tests, were routinely performed to evaluate complications.

### Outcome definitions

Cannulation success was assessed using fluoroscopic images of bile duct cannulation using the catheter or guidewire. The cannulation time for the selected biliary system was calculated by subtracting the time recorded in the endoscopic system at the initial start of the endoscopic cannulation images from the time written on the fluoroscopic images and was available in the proficient nursing assistant chart when the catheter was placed deep in the bile duct. Ease of cannulation was classified as either easy or difficult based on international recommendations that considered a procedure with a cannulation time >5 min, >5 attempts, or >1 main pancreatic duct insertion as difficult [9, 10]. Whether cannulation attempts and insertion of the PD were performed was determined based on the patients’ medical records. PEP was defined according to the Revised Atlanta Classification, which describes abdominal pain and elevation of amylase or lipase levels to three-fold higher than the typical upper limits. Post-ERCP cholangitis was retrospectively investigated by assessing the presence of abdominal pain, fever (>38 °C), and leukocytosis. Post-ERCP bleeding refers to the need for transfusion with the simultaneous presentation of melena or hematochezia. Post-ERCP perforation was defined as the presence of newly developed retroperitoneal or intraperitoneal air on radiological evaluation within 24 h after ERCP.

The primary outcome was the overall success rate of the conventional and new cannulation strategies. Secondary outcomes included cannulation time and post-ERCP complications, especially PEP, in both groups, according to cannulation difficulty. The factors affecting the duration of PCWGC were demonstrated to clarify specific situations in which physicians required more time for cannulation. Furthermore, the success rate and cannulation time of the first and second attempts were analyzed. Subgroup analysis, including cannulation success rate, time, adverse events, and laboratory data based on the cannulation difficulty group, was performed to elucidate the usefulness and safety of PCWGC.

### Propensity score matching (PSM)

The PSM is a method that assigns propensity scores through logistic regression analysis and matches experimental and control groups with similar scores. PSM was applied to balance the covariates between the conventional and new strategy groups. To analyze the outcomes of all cannulation cases for PSM, we first selected age, sex, indication, underlying disease, and laboratory findings as confounding factors, whereas only age and sex were selected as confounding factors for PSM in difficult cannulation cases because of the small number of cases. After performing PSM, conditional logistic regression analysis was conducted to examine the odds ratio of the outcome based on the group variable. The matching algorithm used in this study was nearest neighbor matching with a 1:1 match ratio, and a caliper of 0.2 was set to achieve balance between the groups.

### Statistical analysis

Differences in categorical variables were analyzed using the chi-squared test or Fisher’s exact test. Continuous variables were analyzed using the independent t-test for normality. All p-values (two-tailed) were computed using SPSS software Version 24.0 (IBM, USA) and those <0.05 were considered statistically significant. The odds ratio and 95% confidence intervals for the primary and secondary outcomes of PSM cohort were also calculated by conditional logistic regression analysis. To estimate the factors associated with Jagtome cannulation as a salvage technique, simple linear regression analysis was performed on all continuous covariates, such as laboratory data. After simple linear regression analysis, eight covariances with p-values <0.3 were selected for multivariate regression analysis. The variance inflation factor >10 between two covariances was removed to ensure linearity. Six independent covariates were entered into the multivariate regression analysis using the backward elimination method.

## Results

**Fig 1:**
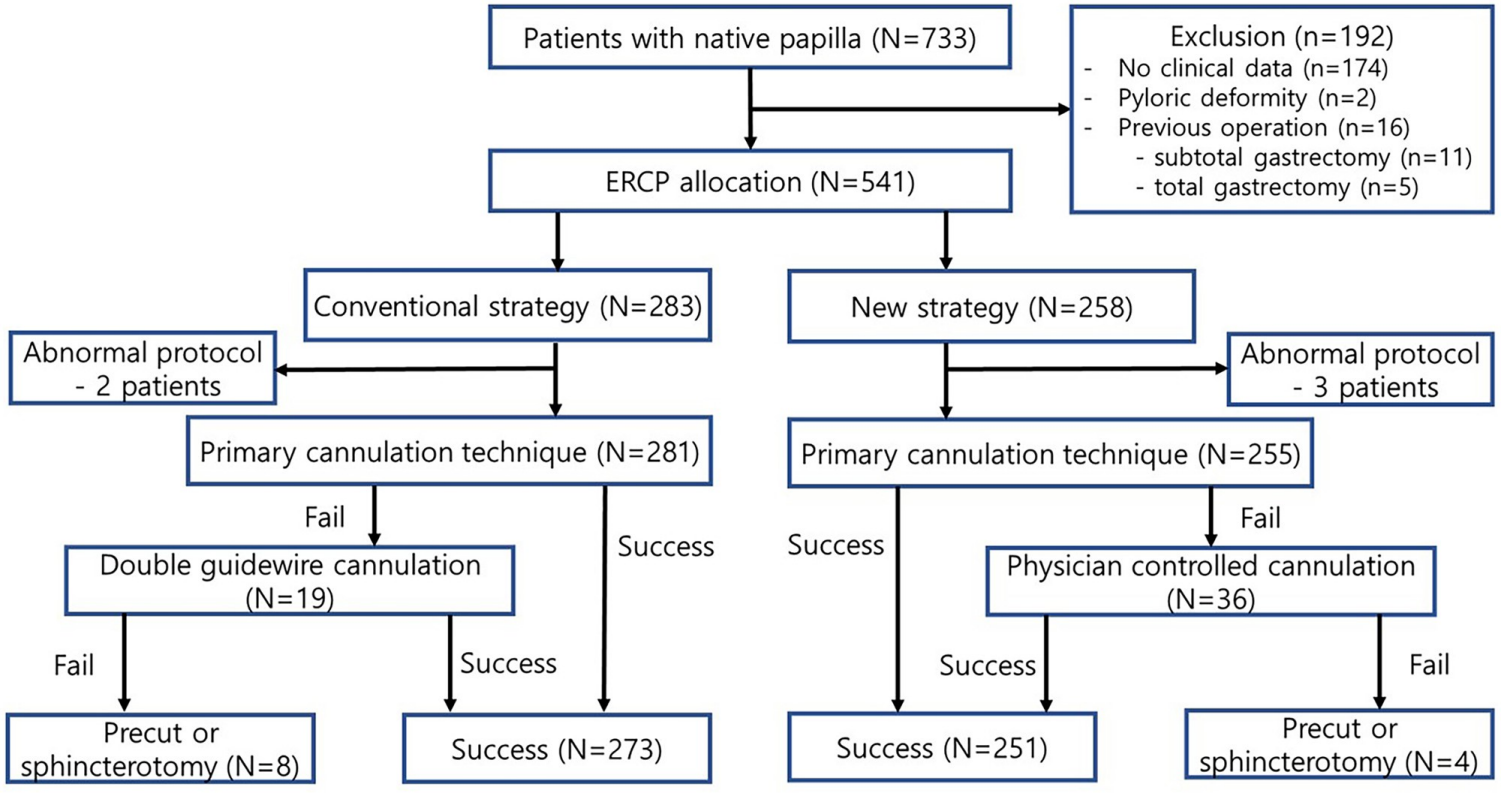
Flowchart of endoscopic retrograde cholangiopancreatography cannulation strategy with clinical results. ERCP, endoscopic retrograde cholangiography and pancreatography.

This investigation included 536 patients, comprising 281 and 255 in the conventional and new strategy groups, respectively, before PSM (Fig 1). Table 1 shows the baseline characteristics of patients who underwent ERCP. The average age of the patients who underwent ERCP was 65.8 and 69.1 years in the conventional and new strategy groups, respectively. The most frequent indication for ERCP was choledocholithiasis in both groups (conventional strategy, 58.7%; new strategy, 58.0%). After 1:1 PSM, both groups comprised 438 individuals (219 in each group). Although age was the only variable with a significant difference, there was no statistically significant difference after PSM (p = 0.887).

**Table 1:** Baseline characteristics of the conventional and new strategy groups with patients with ERCP cannulated before and after propensity score matching.

Before matching			After matching					
Characteristics	Conventional Strategy (N = 281)	New Strategy (N = 255)	P-value	Standardized difference	Conventional Strategy (N = 219)	New Strategy (N = 219)	P-value	Standardized difference
Age (years), Mean(SD)	65.8±15.9	69.1±13.7	0.0111	0.221	68.0±14.7	67.8±14.1	0.887	-0.0136
Sex, n(%)			0.873	-0.0138			0.631	-0.0459
Female	127(45.2)	117(45.9)			97(44.3)	102(46.6)		
Male	154(54.8)	138(54.1)			122(55.7)	117(53.4)		
Indication, n(%)			0.11	0.212			0.942	0.0535
Choledocholithiasis	165(58.7)	148(58.0)			129(58.9)	127(58.0)		
Biliary Benign Stricture	33(11.7)	25(9.8)			26(11.9)	23(10.5)		
Biliary Tumor	50(17.8)	63(24.7)			46(21)	50(22.8)		
Others	33(11.7)	19(7.45)			18(8.2)	19(8.7)		
HTN, n(%)			0.0653	0.16			0.848	0.0183
No	153(54.6)	119(46.7)			106(48.4)	104(47.5)		
Yes	127(45.4)	136(53.3)			113(51.6)	115(52.5)		
DM, n(%)			0.258	0.0979			0.912	-0.0106
No	214(76.4)	184(72.2)			164(74.9)	165(75.3)		
Yes	66(23.6)	71(27.8)			55(25.1)	54(24.7)		
CAD, n(%)			0.348	0.0812			0.894	-0.0127
No	242(86.4)	213(83.5)			185(84.5)	186(84.9)		
Yes	38(13.6)	42(16.5)			34(15.5)	33(15.1)		
Pulmonary, n(%)			0.349	0.0807			0.76	-0.0292
No	273(97.5)	245(96.1)			213(97.3)	214(97.7)		
Yes	7(2.5)	10(3.9)			6(2.7)	5(2.3)		
CVD, n(%)			0.928	-0.0079			0.746	-0.031
No	253(90.4)	231(90.6)			197(90.0)	199(90.9)		
Yes	27(9.6)	24(9.4)			22(10.1)	20(9.1)		
CKD, n(%)			0.427	-0.069			0.507	-0.0634
No	255(91.1)	237(92.9)			197(90.0)	201(91.8)		
Yes	25(8.9)	18(7.1)			22(10.1)	18(8.2)		
Cirrhosis, n(%)			0.799	0.022			0.647	-0.0438
No	266(95)	241(94.5)			208(95.0)	210(95.9)		
Yes	14(5)	14(5.5)			11(5.0)	9(4.1)		
Other malignancy, n(%)			0.289	0.0917			0.787	0.0259
No	245(87.5)	215(84.3)			188(85.8)	186(84.9)		
Yes	35(12.5)	40(15.7)			31(14.2)	33(15.1)		
CRP, mg/L Mean(SD)	36.5±57.4	41.3±61.0	0.344	0.0818	39.8±61.3	39.1±60.8	0.763	-0.029
WBC,10^3^/μL Mean(SD)	8.2±3.7	8.0±4.5	0.547	-0.0519	8.3±3.7	8.0±4.4	0.829	-0.0207
AST, IU/L Mean(SD)	185.0±243.1	162.0±199.8	0.234	-0.104	177.3±242.8	163.6±184.9	0.651	-0.0434
ALT, IU/L Mean(SD)	200.2±224.2	181.2±192.2	0.295	-0.091	193.3±232.5	189.7±198.9	0.46	-0.071
ALP, IU/L Mean(SD)	252.4±218.9	257.9±226.2	0.777	0.0247	249.8±222.6	254.3±217.8	0.971	-0.0034
GGT, IU/L Mean(SD)	375.1±390.8	396.4±450.9	0.558	0.0505	373.5±384.1	388.7±430.0	0.863	0.0166
Direct bilirubin, mg/dL Mean(SD)	2.2±3.2	2.2±3.2	0.835	-0.0181	2.2±3.2	2.2±3.3	0.623	-0.0473
BUN, mg/dL Mean(SD)	15.3±6.7	15.8±7.7	0.4	0.0726	15.9±6.9	15.5±7.1	0.905	0.0115
Cr. mg/dL Mean(SD)	1.0±0.7	0.9±0.5	0.426	-0.0692	1.0±0.8	0.9±0.6	0.814	-0.0226
Amylase, IU/L Mean(SD)	188.2±558.9	175.7±440.5	0.776	-0.0247	162.5±473.4	152.0±374.2	0.798	-0.0245
Lipase, U/L Mean(SD)	499.6±2074.2	375.9±1191.3	0.404	-0.0731	383.0±1613.5	325.5±1063.2	0.660	-0.0421

*, Statistical significance was considered at p < 0.05; SD, standard deviation; COPD, chronic obstructive pulmonary disease; HTN, hypertension; DM, diabetes mellitus; CAD, coronary artery disease; CVD, cerebrovascular disease; CKD, chronic kidney disease; CRP, C-reactive protein; WBC, white blood cell; AST, aspartate transaminase; ALT, alanine transaminase; ALP, alkaline phosphatase; GGT, gamma-glutamyl transferase; BUN, blood urea nitrogen.

The clinical outcomes before PSM are summarized in Table 2. The overall cannulation success rate, including the second cannulation method, was not significantly different between the groups (97.2% vs. 98.4%; p = 0.318). The total cannulation time was similar in both groups (172.3 s vs. 136.3 s; p = 0.051). The initial cannulation method was more successful in the conventional strategy group than that in the new strategy group (93.2% vs. 85.9%, p = 0.005), and there was statistically significant difference in the initial cannulation time (p = 0.0152). Rescue management failed to achieve successful cannulation in eight patients (2.9%) in the conventional strategy group and in four patients (1.6%) in the new strategy group. The incidence of PEP in the conventional and new strategy groups was 1.8% and 2.4%, respectively, indicating no significant difference between the two groups.

**Table 2:** Comparison of clinical outcomes between conventional and new strategy groups in the before PSM and after PSM cohorts.

	Before PSM cohort			After PSM cohort		
	Conventional strategy (N = 281)	New strategy (N = 255)	P-value	Conventional strategy (N = 219)	New strategy (N = 219)	P-value
Total cannulation success rate, n(%)	273 (97.2)	251 (98.4)	0.318	210 (96.8)	215 (99.1)	0.0921
Total cannulation time, mean(SD)	172.3±240.5	136.3±171.7	0.051	177.7±246.6	140.6±182.1	0.0779
Primary cannulation success rate, n(%)	262/281 (93.2)	219/255 (85.9)	0.005*	201 (92.6)	186 (85.7)	0.0205*
Primary cannulation time, mean(SD)	154.3±225.7	113.1±118.6	0.0152*	157.0±228.7	113.7±123.6	0.0225*
Secondary cannulation success rate, n(%)	11 (3.9)	32 (12.6)	0.0002*	9 (4.2)	29 (13.4)	0.0007*
Secondary cannulation time, mean(SD)	600.7±183.9	295.1±330.2	0.0059*	639.7±175.9	313.0±342.1	0.0096*
Types of salvage techniques						
None	262 (93.2)	219 (85.9)		201 (92.6)	186 (85.7)	
Double guidewire technique	11 (3.9)	0		9 (4.2)	0	
Physician-controlled wire-guided cannulation	0	32 (12.6)		0	29 (13.4)	
Precut infundibulotomy or sphincterotomy	8 (2.9)	4 (1.6)		7 (3.2)	2 (0.9)	
Complications						
Post-ERCP pancreatitis, n(%)	5 (1.8)	6 (2.4)	0.64	3 (1.4)	6 (2.7)	0.312
ERCP-related bleeding, n(%)	1 (0.4)	2 (0.8)	0.507	0	1 (0.5)	0.317
ERCP-related infection, n(%)	17 (6.1)	15 (5.9)	0.935	12 (5.5)	13 (6.0)	0.837
ERCP-related perforation, n(%)	0	0	N/A	0	0	N/A

*, Statistical significance was considered at p < 0.05; CI, confidence interval; N/A, not applicable; ERCP, endoscopic retrograde cholangiopancreatography; SD, standard deviation.

We conducted PSM to reduce selection bias in both groups. In the PSM cohort, the total cannulation success rate was similar between the groups (96.8% vs. 99.1%), and there was no significant difference in the total cannulation time (p = 0.0779). Specifically, the primary cannulation success rate was significantly lower in the new strategy group than that in the conventional strategy group (85.7% vs. 92.6%). However, PCWGC showed a higher secondary cannulation success rate (13.4% vs. 4.2%) and a shorter cannulation time than DGT (131.0 s vs. 639.7 s). There was no significant difference in the occurrence of PEP between the two groups.

Before PSM cohort in the difficult cannulation subgroup showed insignificant overall success rate (65.2% vs. 87.2%) (Table 3). The primary success rates were 24% and 18.2% in the conventional and new strategies, respectively. The salvage techniques revealed significant differences in success rates between the two strategies (conventional strategy; 11/19, 44.0% vs. new strategy; 32/36, 72.7%, p = 0.0179). The total cannulation time in the new strategy group (using PCWGC) was remarkably shorter than that in the conventional strategy group (using DGT) (656.8 s vs. 293.2 s). The primary and salvage cannulation time in the new strategy was significantly shorter than that in the conventional strategy group. The incidence of PEP was 4.5% (2/44) and 12.0% (3/25) in the new and conventional strategies, respectively. Post-intervention amylase and lipase levels in both groups were not significantly different.

**Table 3:** Subgroup analysis of the outcomes between two strategies in the difficult cannulation group.

	Before PSM cohort			After PSM cohort		
	Conventional strategy (N = 25)	New strategy (N = 44)	p-value	Conventional strategy (N = 23)	New strategy (N = 23)	p-value
Total cannulation success rate, n(%)	17 (68)	40 (90.9)	0.0158*	15 (65.2)	20 (87.0)	0.0839
Total cannulation time, Mean(SD)	656.8±353.8	293.2±304.2	0.0002*	684.3±366.6	354.3±348.2	0.0105*
Primary cannulation success rate, n(%)	6 (24)	8 (18.2)	0.564	6 (26.1)	2 (8.7)	0.120
Primary cannulation time, Mean(SD)	759.8±559.8	285.4±180.7	0.0423*	759.8±559.8	355.5±183.1	0.375
Salvage cannulation success rate, n(%)	11 (44)	32 (72.7)	0.0179*	9 (39.1)	18 (78.3)	0.007*
Salvage cannulation time, Mean(SD)	600.6±183.9	295.1±330.2	0.0059*	634.0±179.5	354.1±365.4	0.0409*
Complications						
Post-ERCP pancreatitis, n(%)	3 (12.0)	2 (4.5)	0.344	3 (13.0)	2 (8.7)	0.636
ERCP-related bleeding, n(%)	1 (4)	1 (2.3)	0.681	1 (4.4)	0	0.312
ERCP-related infection, n(%)	3 (12)	3 (6.8)	0.463	3 (13.0)	1 (4.4)	0.295
ERCP-related perforation, n(%)	0	0	N/A	0	0	N/A
Laboratoy						
Post-amylase, IU/L Mean(SD)	297.9±468.2	235.4±362.2	0.538	305.3±488.2	233.9±327.1	0.563
Post-lipase, U/L Mean(SD)	564.2±1047.8	422.0±935.9	0.563	599.4±1086.7	406.7±774.0	0.492
Δ amylase, IU/L Mean(SD)	219.0±468.9	48.7±583.4	0.216	229.6±488.1	27.6±628.0	0.230
Δ lipase, U/L Mean(SD)	474.5±1053.7	5.8±1456.1	0.163	505.0±1094.6	-24.6±1448.4	0.169

*, Statistical significance was considered at p < 0.05; CI, confidence interval; CI, confidence interval; SD, standard deviation; ERCP, endoscopic retrograde cholangiopancreatography.

In the PSM group, there was no statistically significant difference in the total and primary cannulation success rates between the two groups. However, the salvage cannulation success rate was significantly higher for PCWGC used in the new strategy group than that for DGT used in the matched difficult biliary cannulation group (9/23, 39.1% vs. 18/23, 78.1%; p = 0.007). No significant differences in complications, including PEP, were observed between the two groups (13.0% vs. 8.7%).

Table 4 displays the outcomes derived from the PSM analysis, which was conducted to reduce selection bias, on crucial outcome variables identified in Tables 2 and 3. Table 4 shows the rates of cannulation success and the odds of PEP based on the strategies employed. After PSM, the primary cannulation success rate for both easy and difficult cases significantly decreased by 46.7% in the new strategy group compared with that in the conventional strategy group. However, the salvage cannulation success rate in the new strategy group significantly increased by 2.889 times (95% CI [1.354–6.165]) compared to that in the conventional strategy group. There was no significant difference in the incidence of PEP between groups. In contrast, in the difficult biliary cannulation group after PSM, the salvage cannulation success rate of PCWGC was four times higher in odds ratio than that of DGT in the difficult biliary cannulation group after PSM (95% CI [1.129–14.175]).

**Table 4:** Odds ratios for cannulation success rate and post-ERCP between the new strategy and the conventional strategy.

	New strategy (vs. Conventional strategy)	Number of cases	Odds ratio (95% CI)	P-value
Easy + Difficult cannulation case	Overall cannulation success rate	427	1.75 (0.512–5.978)	0.372
	Primary cannulation success rate	392	0.533 (0.291–0.978)	0.0423*
	Salvage cannulation success rate	35	2.889 (1.354–6.165)	0.0061*
	Post-ERCP pancreatitis	9	2 (0.5–7.997)	0.327
Difficult cannulation case	Overall cannulation success rate	35	3.5 (0.727–16.848)	0.118
	Primary cannulation success rate	8	0.333 (0.067–1.652)	0.179
	Salvage cannulation success rate	27	4 (1.129–14.175)	0.0317*
	Post-ERCP pancreatitis	5	0.5 (0.045–5.514)	0.571

*, Statistical significance was considered at p < 0.05; CI, confidence interval; ERCP, endoscopic retrograde cholangiopancreatography.

Table 5 shows the factors that influence cannulation time, based on model prediction. The PCWGC cannulation time was affected by three factors; CRP, direct bilirubin, and lipase. With an increase in CRP (mg/L), direct bilirubin (mg/dL), and lipase (U/L) levels by 1, the cannulation time increased by 0.326, 0.453, and 0.518 s, respectively. The coefficient of determination (R^2^) of the multiple regression model with these three factors was 42.3%, indicating that the endoscopist’s Jagtome cannulation time varied depending on the bile duct status presented by inflammation, obstruction, and pancreatitis.

**Table 5:** Multiple regression model affecting physician-guided cannulation time (N = 32).

	β	p	VIF
CRP, mg/L	0.326	0.025*	1.017
Direct bilirubin, IU/L	0.453	0.003*	1.054
Lipase, U/L	0.518	0.001*	1.049

*, Statistical significance was considered at p < 0.05; CRP, C-reactive protein; VIF, variance inflation factor.

## Discussion

Safe cannulation is an indispensable prerequisite for the most advanced ERCP techniques. Typically, selective biliary system cannulation comprises a two-stage process that includes a shallow catheter or sphincterotome tip cannulation of the ampulla orifice and further deep cannulation of the bile duct. Cannulation, being a sophisticated procedure, has been documented to increase the incidence rate of PEP from 2.8% to 11.5% when performed following the conventional methods, depending on the clinical difficulty of the case, which is also a known independent risk factor [11]. Therefore, relatively safer and more efficient cannulation techniques are required. Although the international consensus recommends alternatives for difficult cannulation [12], the fact that there are no established guidelines for salvage cannulation methods after a failed initial attempt emphasizes the need for a retrospective chart review.

According to a recent network model meta-analysis, the cannulation success rate of TPS was the highest (p = 0.97) [13]. According to the head-to-head comparison analysis, the cannulation success rate of TPS was 1.29 times higher (95% CI, 1.05–1.59) than that of the conventional standard cannulation method, and 1.21 times higher (95% CI, 1.01–1.44) than that of DGT. The PEP rate of the early needle-knife technique was the lowest among the methods evaluated, showing a 39% decrease compared to standard cannulation, and thus being the most superior. In addition, as mentioned earlier, TPS resulted in a 47% reduction in the PEP rate compared to DGT (95% CI, 0.30–0.92). The aforementioned meta-analysis did not compare PCWGC with other methods, making it difficult to confirm its superiority over other existing methods. However, the strength of this study is that it issued the salvage technique for PCWGC by comparing it with DGT. Further prospective studies comparing PCWGC with other methods using arm-to-arm comparisons are required.

The current retrospective study, designed as a two-arm, single-center study, compared the clinical efficacy of salvage techniques, PCWGC and DGT, with the assistance of nurses. In our analysis after PSM, PCWGC was chosen more frequently in the difficult cannulation subgroup, with a success rate of 87.0% and cannulation time of 354.3 s. The incidence of PEP demonstrated no statistically significant difference between the groups, which is suggestive of an efficient cannulation method with few complications and favorable outcomes. The overall findings suggest that PCWGC, which proceeds without an assistant, may increase cannulation efficacy to achieve success in difficult cannulations that fail in more than five attempts. The above results should have been selectively adopted in difficult cannulations (5<attempts<15) because DGT is known to be effective in cases with >15 failed attempts [14]. Although the definition of difficult cannulation remains open to discussion and may require further investigation, PCWGC could be a good alternative in cases of difficult cannulation.

Although the differences between the two groups in the overall success rate and total cannulation time were not significant (96.8% vs. 98.2%; p = 0.0360; 174.4 s vs. 136.9 s, p = 0.0728), PCWGC on the second attempt proved its superiority over DGT as a salvage technique in terms of success rate and cannulation time (78.3% vs. 39.1%, p = 0.007; 354 s vs. 634 s, p = 0.0409). Previous reports comparing DGT with conventional cannulation for difficult cannulation documented no significant difference in the cannulation success rate. Although there is a limitation in that PCWGC is superior to DGT in a direct comparison because of the differences in the devices used, such as sphincterotomes and guidewires, the success rate of PCWGC was higher than that of DGT (78.3% [18/23] vs. 39.1% [9/23]). Based on a comparison with other studies that reported a success rate of DGT of 56.4% [15], which is slightly higher than that of DGT in our study, PCWGC could result in a better success rate than DGT in a 1:1 comparison [16]. The primary cannulation success rate in the PCWGC group was 85.7%, which was significantly lower than that in the conventional cannulation group (p = 0.005). Although we predicted that the primary success rates in both groups would be similar, the following interpretation could be a result of the difficulty of cannulation in the PCWGC group (44/255, 17.3%), which was two-fold higher than that in the conventional group (25/281, 8.9%). To reduce the group differences in the difficulty cannulation case mentioned earlier, PSM was performed. In Table 4, the primary cannulation success rate (26.1% vs 8.7%) showed no significant difference between both groups. Moreover, odds ratios of the primary cannulation success rate for difficult cannulation cases showed no difference between the two groups, as demonstrated by the findings in Table 5 (p = 0.179). Thus, the efficacy of PCWGC as an additional salvage technique is highlighted by its success rate and cannulation time.

A literature review revealed that failed selective bile duct cannulation could occur in 5–15% of cases, even if a skilled endoscopist conducts the procedure [17]. Records from another study documented that the cannulation success rate of ERCP was >95% at a referral center. A recent prospective randomized controlled trial by Buxbaum et al. compared the efficacy and safety of PCWGC with assistant-controlled wire-guided cannulation [18]. According to this study, the biliary cannulation success rates (<eight attempts) of the endoscopist- and assistant-controlled procedures were 89.9% and 89.7%, respectively. The initial biliary cannulation success rates were similar between the two groups (74.3% vs. 74.8%). A single-center prospective study reported that the success rate of initial selective bile duct cannulation using PCWGC within 10 min was 72.5% [8]. In our retrospective study, the success rate of total cannulation was 97.2–98.4%, and the primary cannulation rate was 85.9–93.2% higher than that reported in previous studies. In cases of difficult cannulation, the initial success rate of cannulation accounted for 18.2–24.0% in both groups (p = 0.564). The gap between the salvage techniques ranged from 57.9% to 88.9% (p = 0.015, 95% CI 8.6–57.3), which suggests the establishment of PCWGC as an alternative technique for failed initial cannulation.

A multicenter randomized controlled trial demonstrated that DGT is not superior to standard cannulation. However, it can be replaced with the standard method when multiple attempts are required and unintended pancreatic duct cannulation occurs [19, 20]. The results of the present study showed that single-operator-guided cannulation as a salvage technique was superior to DGT in terms of success rate and cannulation time. This implication could be attributed to the advanced short guidewire system that allows the exclusion of the need for communication between assistants, thereby enabling endoscopists to manipulate the guidewire placement and respond quickly to an uncomfortable feeling of cannulation.

Cannulation is the first manipulation of the bile duct, which limits the remaining ERCP procedure and influences the incidence of PEP. The most common benchmark of adverse events after ERCP is PEP, with an incidence rate of 3.5% [21]. Our study reported an overall PEP rate of 2.1% (11/536). Furthermore, the incidence of PEP using the single-operator wire-guided cannulation technique was 2.4%, which was lower than that reported in other studies [22]. The rate of PEP in the difficult cases increased to 13.0% in the conventional strategy group and 4.5% in the single-operator strategy group, which is consistent with the findings of a multicenter prospective study that reported a PEP rate between 4.3% and 11.3% [23]. Another single-center study reported rates ranging from 3.3% to 14.9% [24]. Thus, our results confirm that, although PCWGC is a promising salvage technique, the PEP rate increases in cases of difficult cannulation, which is consistent with the findings of other studies that investigated the incidence rates of PEP depending on cannulation difficulty.

The incidence of PEP associated with cannulation difficulty increases with prolonged cannulation attempts and duration [11, 25]. The results of our study contradict those of previous studies related to cannulation, which reported that the incidence rate of PEP increases with cannulation difficulty. The results obtained in this investigation indicate that the PEP rate in the PCWGC group did not significantly increase with cannulation difficulty (1.9% vs. 4.5%, p = 0.277) (S1 Table). This could be attributed to the reduced sustained trauma, as there was no need to proceed with an additional guidewire, and the hydrophilic surface covering the nitinol core of the guidewire enhanced its pushability and flexibility, leading to reduced bile duct injury.

This study strengthens several previous findings, as our results indicate that PCWGC was superior to salvage therapy on the second attempt in terms of success rate and total cannulation time. Further, the investigation demonstrated no differences in the PEP incidence between the two groups, although meta-analysis on DGT for PEP prevention reported increased PEP than other advanced techniques (RR, 1.98, 95% CI 1.14–3.42) [16]. Half as many pre-cuts were performed in the conventional group compared with those in the PCWGC group (8, 2.8% vs. 4, 1.6%), suggesting that the new strategy may prevent PEP and reduce the need for invasive advanced procedures. Another remarkable finding is the data of staged success rates related to PCWGC and DGT, which allow the comparison and clarification of the advantages of PCWGC. Finally, the study evaluated the difference between the before and after values of amylase and lipase to evaluate the safety of PCWGC, which suggested that the staged cannulation scheduled for PCWGC in the second trial was clinically superior to the conventional strategy. While PCWGC cannot be recommended to ERCP beginners with little experience as it is a salvage technique performed without an assistant, it might be considered as a primary or secondary cannulation method for skilled endoscopists.

However, this study has several limitations, mostly stemming from its small sample size and retrospective design. The main issue raised and unresolved in this study was whether the cannulation success rate and time of PCWGC could be affected by anatomical variants. The cannulation success rate and time are influenced by the morphology of the ampulla, juxtapapillary structures, and presence of anomalies [26–28]. Our analysis contained limited information regarding the anatomical factors of the biliary system, which restricted the analysis. Specifically, the present retrospective study outlined a nonrandomized observational design that inevitably included an unintended selection bias in the distribution of difficult cannulation groups, leading to limited generalization of the results. To minimize selection bias, we performed PSM to analyze the results and obtained the same results. However, as not all matching variables were used owing to the small sample size of difficult cannulation cases, further prospective clinical trials are warranted to overcome this issue. Another limitation is the time bias caused by differences in endoscopist experience. The endoscopist of the new strategy group was more experienced and more skilled in cannulation than the endoscopist of the conventional strategy group because of the PCWGC, which was implemented after 2019. Because four endoscopists with various levels of ERCP experience in a single center conducted the PCWGC procedure, the study lacked consistent determination and corroboration in the assessment of procedure difficulty. Another limitation of our study was the presence of errors originating from the calculation of cannulation time using endoscopic and fluoroscopic images, which could have resulted in inaccurate results. The DGT and precut techniques are more advanced techniques used for difficult cannulation than PCWGC. Thus, the cannulation time was longer in the conventional strategy group than that in the new strategy group.

## Conclusions

In summary, we retrospectively detailed our experience with biliary system access strategies according to cannulation difficulty and staged cannulation methods. PCWGC, as a salvage technique, achieved better efficacy, originating from a remarkably high success rate and improved cannulation time with a similar PEP rate, compared to the conventional strategy in the difficult cannulation subgroup. Given the limited circumstances and personal resources, PCWGC should be considered as an alternative rescue method in cases requiring repeated cannulation.

## Supporting information

S1 Table Comparison of primary clinical outcomes before propensity score matching cohort between easy and difficult groups based on strategy. (DOCX)
