# Supplementary material for: Propensity score-matched analysis of physician-controlled wire-guided cannulation as an effective technique against difficult cannulation in endoscopic retrograde cholangiopancreatography: A retrospective study
Source: PLoS One. 2023 Apr 28;18(4):e0285118. doi: 10.1371/journal.pone.0285118 (PMC10146477; doi:10.1371/journal.pone.0285118)
Supplement: S1 Table — (DOCX) [file pone.0285118.s001.docx]

S1 Table. Comparison of primary clinical outcomes before propensity score matching cohort between easy and difficult groups based on strategy

|  | Conventional strategy group (N=281) | | | New strategy group (N=255) | | |
| --- | --- | --- | --- | --- | --- | --- |
|  | Easy  (N=256) | Difficult  (N=25) | p-value | Easy  (N=211) | Difficult  (N=44) | p-value |
| Total cannulation success rate†  (n, %) | 256/256  (100) | 17/25  (68.0) | < 0.001* | 211/211  (100) | 40/44  (90.9) | 0.001*^‡^ |
| Total cannulation time†  (median, IQR) | 71  (38.25–148.75) | 626  (425–939) | < 0.001* | 65  (32–144) | 218  (74.75-433.75) | < 0.001* |
| Primary cannulation success rate†  (n, %) | 256/256  (100) | 6/25  (24.0) | < 0.001* | 211/211  (100) | 8/44  (18.2) | < 0.001* |
| Primary cannulation time^§^  (median, IQR) | 71  (38.25–148.75) | 1012  (54.75–1212.5) | 0.029* | 65  (32–144) | 215  (131.75–472.75) | 0.001* |
| Complications |  |  |  |  |  |  |
| Post-ERCP pancreatitis^‡^ (n, %) | 2 (0.8) | 3 (12.0) | 0.006* | 4 (1.9) | 2 (4.5) | 0.277 |
| Post-ERCP bleeding^‡^ (n, %) | 0 (0) | 1 (4.0) | 0.089 | 1 (0.5) | 1 (2.3) | 0.316 |
| Post-ERCP cholangitis^‡^ (n, %) | 14 (5.5) | 3(12.0) | 0.183 | 12 (5.7) | 3 (6.8) | 0.728 |
| Laboratory data |  |  |  |  |  |  |
| CRP^§^, mg/L (median, IQR) | 9.695  (2.203–47.938) | 7.25  (1.795–42.98) | 0.609 | 8.92  (1.83–48.04) | 39.625  (4.918–113.368) | 0.007* |
| Total bilirubin^§^, mg/dL (median, IQR) | 2.265  (0.83–5.143) | 5.46  (1.33–8.085) | 0.079 | 2.01  (0.82–4.31) | 3.5  (1.18–7.97) | 0.016* |
| Direct bilirubin^§^, mg/dL (median, IQR) | 0.9  (0.173–2.935) | 2.49  (0.49–4.735) | 0.058 | 0.74  (0.173–2.268) | 1.5  (0.343–5.055) | 0.018* |
| Amylase^§^, IU/L (median, IQR) | 51.5  (39–78.75) | 65  (49–108) | 0.062 | 53  (40–77) | 31  (16–66) | 0.683 |
| Lipase^§^, U/L (median, IQR) | 32  (15.25–65.00) | 50  (20.50–88) | 0.195 | 64.50  (29.25–112.50) | 49  (16.25–135.50) | 0.132 |
| Post-amylase^§^, IU/L (median, IQR) | 57 (40–108) | 129 (70–199) | < 0.001* | 58 (44–93) | 83 (41–240.50) | 0.052 |
| Post-lipase^§^, U/L (median, IQR) | 46 (25–132.75) | 182 (54.50–539) | < 0.001* | 42 (22–133) | 91.50 (31–262.50) | 0.011* |
| Δ Amylase^§^, IU/L (post-amylase - amylase), (median, IQR) | 0 (-12–17) | 49 (-2–145) | 0.001* | 1 (-13–18) | 5.5 (-29.25–114.25) | 0.156 |
| Δ Lipase^§^, U/L (post-lipase - lipase), (median, IQR) | 5 (-11–28) | 50 (-2–465.5) | 0.001* | 6 (-11–32) | 13.5 (-34.75–134.5) | 0.174 |

*, Statistically significant as P < 0.05; ^†^, Chi-square test, ^‡^, Fisher’s exact test, ^§^, Mann–Whitney test; ERCP, endoscopic retrograde cholangiopancreatography; IQR, interquartile range; CRP, C-reactive protein
